# Optimized Electrode Locations for Wearable Single-Lead ECG Monitoring Devices: A Case Study Using WFEES Modules Based on the LANS Method

**DOI:** 10.3390/s19204458

**Published:** 2019-10-14

**Authors:** Huaiyu Zhu, Yun Pan, Fan Wu, Ruohong Huan

**Affiliations:** 1 College of Information Science and Electronic Engineering, Zhejiang University, Hangzhou 310027, China; 2 Product Department, Hangzhou Proton Technology Co., Ltd., Hangzhou 310012, China; 3 College of Computer Science and Technology, Zhejiang University of Technology, Hangzhou 310023, China

**Keywords:** electrocardiogram monitoring, single-lead, electrode location, QRS amplitude

## Abstract

Body surface potential mapping (BSPM) is a valuable tool for research regarding electrocardiograms (ECG). However, the BSPM system is limited by its large number of electrodes and wires, long installation time, and high computational complexity. In this paper, we designed a wearable four-electrode electrocardiogram-sensor (WFEES) module that measures six-channel ECGs simultaneously for ECG investigation. To reduce the testing lead number and the measurement complexity, we further proposed a method, the layered (*A*, *N*) square-based (LANS) method, to optimize the ECG acquisition and analysis process using WFEES modules for different applications. Moreover, we presented a case study of electrode location optimization for wearable single-lead ECG monitoring devices using WFEES modules with the LANS method. In this study, 102 sets of single-lead ECG data from 19 healthy subjects were analyzed. The signal-to-noise ratio of ECG, as well as the mean and coefficient of variation of QRS amplitude, was derived among different channels to determine the optimal electrode locations. The results showed that a single-lead electrode pair should be placed on the left chest above the electrode location of standard precordial leads V1 to V4. Additionally, the best orientation was the principal diagonal as the direction of the heart’s electrical axis.

## 1. Introduction

Cardiovascular diseases (CVDs) have been a major cause of health loss with high mortality worldwide for a decade. Among CVDs, heart diseases were the leading cause of death [1]. In clinical practice, medical staff diagnose heart diseases mainly through echocardiography, coronary angiography, and electrocardiography (ECG). ECG is the most common and basic diagnostic method for many kinds of cardiac abnormal screening, since it is non-invasive, low-cost, and easy to be applied on patients [2]. The progress in biomedical computing also brought more possibilities for ECG-based cardiology analysis, e.g., myocardial ischemia and infarction detection, arrhythmia identification, and sudden cardiac death evaluation, in which the ECGs with detailed wave morphology features were required [3]. For those studies, the body surface potential map (BSPM) system [4] was a valuable tool recording high-resolution ECGs by measuring bio-potentials from 80 to 200 electrodes on the subject’s chest. The BSPM system was widely applied in cardiology research regarding ECG, such as left circumflex occlusion [5], the personalized cardiac electrophysiology model [6], electrical heterogeneity variety [7], and ECG imaging localization [8]. However, the major disadvantages of the BSPM system are the large number of electrodes and wires, as well as the long installation time needed for measurement preparation [9]. Moreover, it requires high computational complexity for ECG acquisition and analysis.

With the development of mobile health and mobile computing, wearable technologies are changing the field of everyday ECG monitoring. Considering the portable, comfortable, and non-interference requirements, most wearable ECG monitoring devices are designed with limited ECG leads, even a single lead, rather than the standard 12-lead ECG system [10,11,12,13]. Single-lead ECG has been proven to contain substantial biometric information and has been used widely in biomedical diagnoses, e.g., human verification [14], obstructive sleep apnea detection [15], respiratory rate detection [16], and most importantly, arrhythmia detection [17]. With the miniaturization of wearable single-lead ECG monitoring devices, the interelectrode distance of ECG precordial electrodes tends to be small, resulting in a weak ECG signal [18]. Furthermore, the orientation of the leads also affects the morphology and amplitude of the measured ECG. Therefore, the identification of the optimal location and orientation for an electrode pair has become an important ECG research topic.

BSPM systems were studied to determine the optimized single-lead ECG location as well. Puurtinen et al. [19] and Donnelly et al. [20] analyzed the BSPM data to locate a site that offered a reliable ECG signal. Lux et al. [21] used the BSPM system for studying the single-lead site where the P wave had the maximal magnitude. Puurtinen et al. [22] also studied 120 single leads from a BSPM system to find the optimal location for a closely located bipolar electrode pair. Väisänen et al. [23] used the BSPM to investigate the optimal electrode location that provided good performance in differentiating left ventricular hypertrophy from the normal. As other ECG investigations with BSPM systems, this research still suffered from complex electrodes, wires, installation, and analysis complexity of BSPM systems.

In this paper, we developed a wearable four-electrode ECG-sensor (WFEES) module as an ECG investigating tool, which could record ECG data from six single-lead channels simultaneously. We further proposed a novel method, the layered (*A*, *N*) square-based (LANS) method, to optimize the ECG investigating process with the WFEES modules. The LANS method is a two-step ECG testing protocol. It realized a tradeoff between experimental time and space according to different applications and the number of WFEES modules. Meanwhile, we designed a case study applying the WFEES modules and the LANS method to find the optimal electrode locations for wearable single-lead ECG monitoring devices efficiently. In this study, 19 healthy subjects (7 females and 12 males) were tested in a quiet and peaceful state. The signal-to-noise ratio (SNR) and QRS amplitude of 102 single-lead ECG data were analyzed, and the optimal electrode locations, as well as the best orientation of electrode pairs, were determined for single-lead wearable ECG monitoring devices. Based on the self-designed WFEES module, the proposed LANS method could significantly reduce the computational complexity for optimal electrode location determination compared to the traditional BMSP system.

## 2. Materials and Methods

### 2.1. WFEES Module

**Figure 1:**
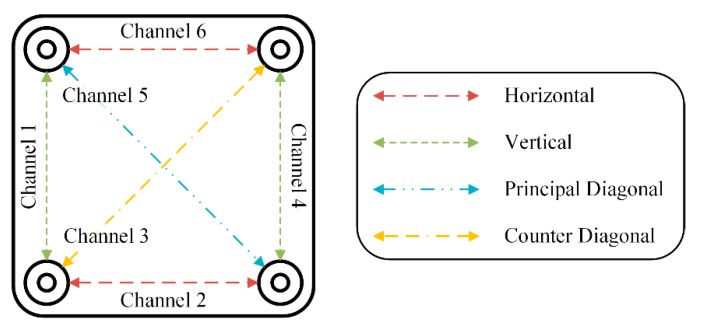
Simplified diagram of the wearable four-electrode electrocardiogram-sensor (WFEES) and its channels’ orientation.

**Figure 2:**
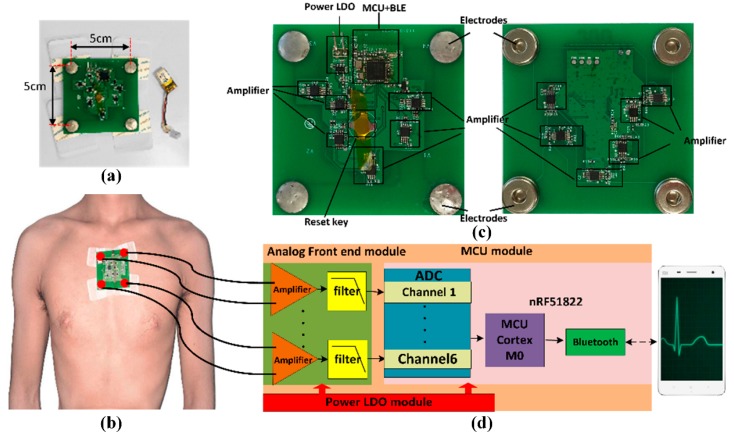
System overview: (**a**) The system look; (**b**) The system mounted on the body of a subject; (**c**) Front and back views of the circuit board; (**d**) Schematic diagram of the hardware.

We designed the WFEES module to record six channels of single-lead ECG signals. Each permutation of two electrodes can be seen as a single-lead channel. That is, there were six single-lead channels, and the ECG signal obtained from each channel was the difference between the two electrodes. Figure 1 showed a simplified diagram of the WFEES module. It illustrated how the six single-lead channels were arranged and their orientation. The system could record six-channel ECG data at the same time, with two horizontal, two vertical, and two diagonal leads. The system overview was shown in Figure 2. Figure 2a presents the appearance of the WFEES system. Previous studies proved that a 5 cm distance between electrodes could provide a reliable ECG signal [18,22]. Thus, the distance between the two electrodes was set to 5 cm. Figure 2b illustrates how the WFEES module was worn on a test subject in one location. The system was attached to the subject’s body using four disposable Ag/AgCl electrodes. The front and back views of the circuit board are shown in Figure 2c. The system architecture of the hardware is shown schematically in Figure 2d.

The WFEES was powered by a rechargeable 3.7 V lithium–ion battery. The analog front end adopted six differential circuits, each of which used two-stage amplifiers: One instrumentation amplifier, i.e., INA321, and one operational amplifier, i.e., OPA2333, resulting in a total magnification of 1000 V/V. The microprocessor control unit (MCU) module used an nRF51822 chip equipped with a low-power ARM Cortex-M0 core. The embedded 2.4 GHz transceiver supported both Bluetooth 4.0 Low Energy (BLE) and the Nordic Gazell 2.4 GHz protocol stacks. The sampling rate for ECG data was 200 Hz. The six-channel ECG data were packed and transmitted to an Android application via Bluetooth every 7.5 ms. Once the test was completed, the data stored in the smartphone’s memory were imported to a PC for further processing. Table 1 summarizes the key parameters of the WFEES system.

### 2.2. LANS Method

#### 2.2.1. Torso Segmentation

**Figure 3:**
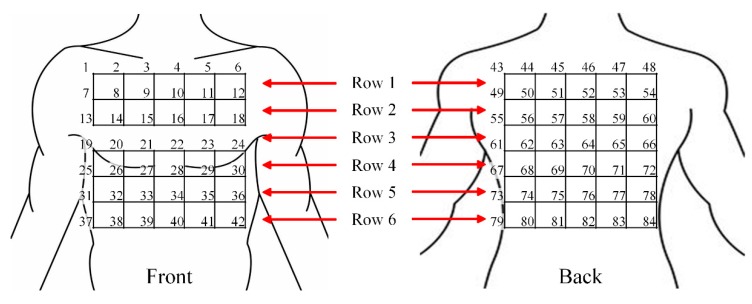
Torso segmentation for the front and back side of the human body.

Previous studies showed that a 5 cm distance between electrodes provided an acceptable SNR [16,24], and 30 to 32 electrodes were sufficient for recording ECG signals [25]. According to the average size of a Chinese adult [26], the average shoulder breadth is 37 cm and the hip breadth is 30 cm on average. Accordingly, we arranged 42 electrode locations on the front and back side of the human torso, respectively, marked uniformly with numbers from 1 to 84 in our study, as shown in Figure 3. Each pair of numbers represented a location and orientation of a channel. Every four adjacent electrodes formed a square with a side length of 5 cm, to which the WFEES module could be attached. Due to the physiological structure of the body, the front side of the torso may not be a plane. So we optimized the squares for better adaptation to the human body, as the squares of row 3 were only arranged on the back side of the torso, for later ECG data analysis. As a result, there were 25 squares and 30 squares on the front and back side of the human torso, respectively. The WFEES modules could be mounted on those squares, and we could record a set of six single-lead ECG data from each module.

#### 2.2.2. Layered (A, N) Square-based Method

**Figure 4:**
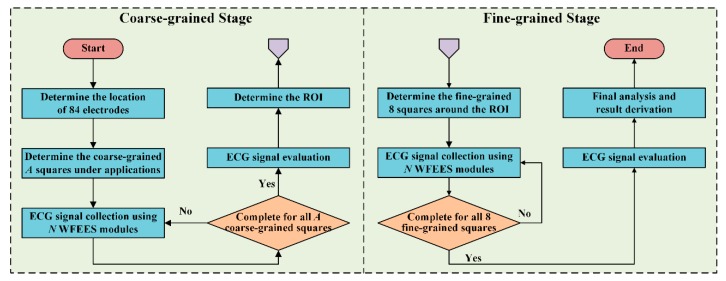
The block diagram of the layered (*A*, *N*) square-based (LANS) method, including the coarse-grained stage and the fine-grained stage.

To reduce the ECG investigation complexity using the WFEES modules, we proposed a novel layered (*A*, *N*) square-based method, i.e., LANS method, which included two stages, i.e., the coarse-grained stage and the fine-grained stage. Here, the “*A*” in LANS stands for the Application-based coarse-grained requirement, in order to find the region of interest (ROI) for a certain ECG application efficiently; and the “*N*” stands for the Number of WFEES modules, which also represents a tradeoff between experimental time and space for ECG research. The process of the coarse-grained stage and fine-grained stage is revealed in Figure 4.

**Figure 5:**
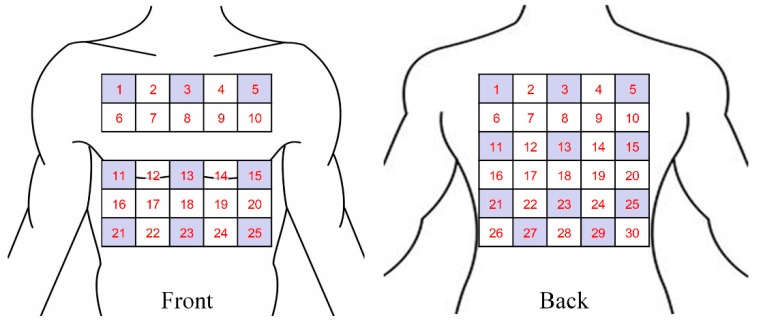
Schematic diagram of squares and the locations of coarse-grained *A* squares, *A*_max_ = 20 (dark areas).

In the coarse-grained stage, we selected several squares, i.e., *A* squares, of which the locations were determined by different applications of ECG research. By computing the differences between signals from any two electrodes, a total of *N* WFEES modules could record *N* sets of six-channel ECG data simultaneously for all *A* squares with ⌈*A*/*N*⌉ rounds, where the “⌈ ⌉” symbol referred to the ceiling function; thus, *A* × 6 single-lead ECG data could be collected during the coarse-grained stage. The schematic diagram of squares for the front and back side of the human body is given in Figure 5. Note that the LANS method contained the following fine-grained stage to collect ECGs around coarse-grained *A* squares, so these *A* squares should not be adjacent. Therefore, the maximum value of *A* was 20, of which the locations are shown as the dark areas in Figure 5. The ECG data of the coarse-grained stage were then processed and evaluated, so the square, of which the ECG signals were relatively better than others for a predetermined purpose, i.e., ROI, could be determined.

**Figure 6:**
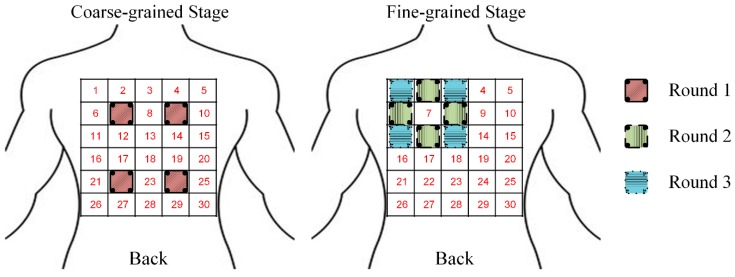
Example of electrocardiography (ECG) investigation using WFEES modules based on L44S method with three rounds.

In the fine-grained stage, we selected eight square locations around the ROI. As a result, nine square locations, the ROI and eight new square locations, were marked. We then repeated the ECG recording and collecting procedure in the coarse-grained stage using *N* WFEES modules, and evaluated the ECG data acquired from these eight new squares, with ⌈8/*N*⌉ rounds. Finally, the reasonable ECG leads for a certain research design could be identified, as the optimal locations and orientations were derived by the LANS method. Figure 6 presents an example of ECG investigation using four WFEES modules with four coarse-grained squares, i.e., by applying the L44S method. We first assigned squares 7, 9, 22, and 24 on the back side in Figure 5 as the application-based coarse-grained squares, and applied four WFEES modules to collect ECGs of these squares simultaneously. Assuming square 7 was the ROI after ECG evaluation, we further marked squares 1, 2, 3, 6, 8, 11, 12, and 13 as the fine-grained squares, and tested them using four WFEES modules within two rounds. As a result, the optimal ECG leads for the application could be derived within three test rounds.

### 2.3. Case Study: Optimized Electrode Locations Determination

#### 2.3.1. Experimental Design

Wearable single-lead ECG monitoring devices play a more and more significant role in everyday ECG analysis for both personal healthcare and clinical evaluation. Hence, it is important to determine the optimal ECG lead location for those devices, for the purpose of enabling them to collect ECG signals that are more conducive to arrhythmia analysis, which is the major usage of wearable single-lead ECG monitoring devices. As the SNR and QRS waveform of ECGs are key indicators for arrhythmia detection, we used three ECG evaluation indices, i.e., SNR, average QRS amplitude, and the coefficient of variance (CV) of QRS amplitudes, to determine the optimal electrode location. The QRS amplitude was defined as the difference between the value at the R peak and the minimum value in the Q and S peaks. The SNR and CV were derived with Equations (1) and (2), respectively.

(1) $$SNR=10\log_{10}\left(\frac{power\;of\;signal\;}{power\;of\;noise}\right)$$

(2) $$CV=\frac{standard\;deviation\;of\;QRS-complex\;amplitude\;}{average\;value\;of\;QRS-complex\;amplitude}\times 100\%$$

**Figure 7:**
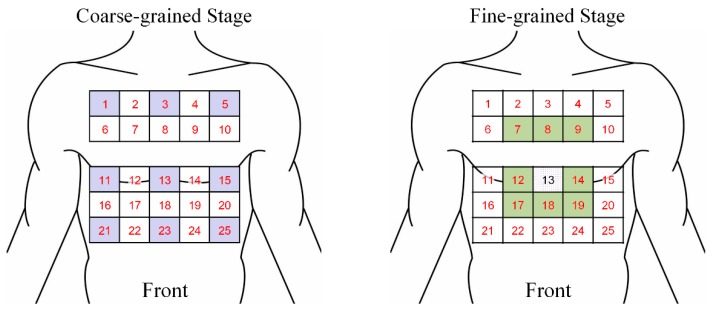
Optimized electrode locations determination using one WFEES module based on the L91S method.

In this case study, we evaluated ECG signals using one WFEES module with the L91S method, that is, we selected nine squares in the coarse-grained stage. Considering the common wearing protocol of wearable single-lead ECG monitoring devices, these squares, i.e., squares 1, 3, 5, 11, 13, 15, 21, 23, and 25, were all distributed on the front side of the human body, as the blue squares shown in Figure 7. Thus, a total 54 single-lead ECG data sets could be collected and analyzed in the coarse-grained stage to determine the ROI. In the fine-grained stage, assuming that the ROI was square 13, eight new squares, i.e., squares 7, 8, 9, 12, 14, 17, 18, and 19, would be arranged around it, as the green squares shown in Figure 7. With the method described, we had a total of 17 squares needed to be assessed, indicating that only 17 measurement sets were required for each subject. Then, ECG data for a total of 102 leads, i.e., 34 horizontal, 34 vertical, 17 principal diagonal, and 17 counter diagonal ECG leads, were recorded and evaluated to identify the optimal location to place single-lead ECG monitoring devices.

#### 2.3.2. Study Population

In this case study, 19 young (average age of 24.6 years), healthy subjects (12 males and 7 females) with no evidence of cardiovascular disease volunteered to participate. The WFEES was mounted on each square determined in Section 2.3.1, and we recorded 1 minute of ECG data for each square on every subject, and evaluated signal quality offline. During the experiment, all subjects were required to keep calm and sit in a chair to keep the skin–electrode interface stable.

#### 2.3.3. Signal Processing

**Figure 8:**
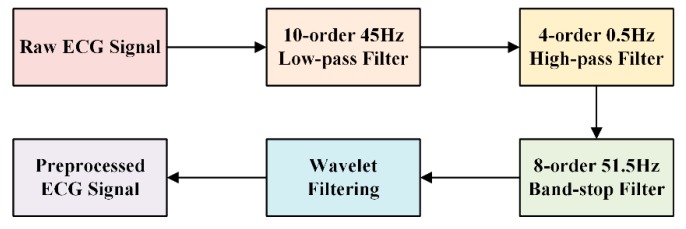
Block diagram of ECG preprocessing.

**Figure 9:**
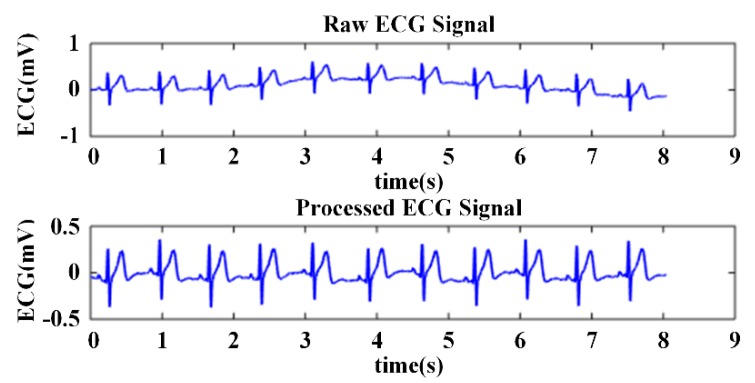
Comparison of the raw and preprocessed ECG signal.

Although the ECG data collected by the WFEES module were passed through the hardware filter, there was still some interference affecting the quality of the signals. Baseline wandering caused by individual movement or breathing may induce a notable drift trend in raw ECG data. Consequently, we used the infinite impulse response (IIR) Butterworth filtering and wavelet filtering method to process the raw ECG data, as shown in Figure 8. After the IIR filtering, an eight-layer “bior3.5” wavelet decomposition was applied on the raw ECG data sets [27]. The baseline wandering and the low-frequency noises were eliminated by setting the level 8 approximation coefficients to zero. Other high-frequency noises were eliminated by thresholding the detail coefficients. Figure 9 presents the performance of ECG preprocessing. The ECG signal became smooth and stable for further feature extraction.

**Figure 10:**
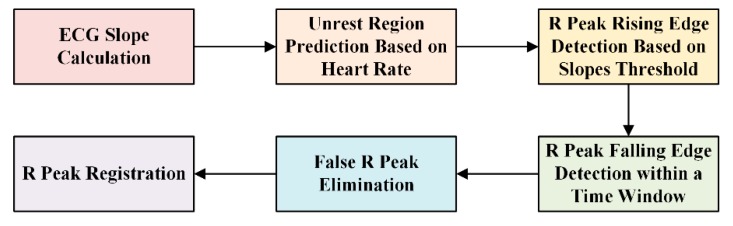
Block diagram of R peak detection.

**Figure 11:**
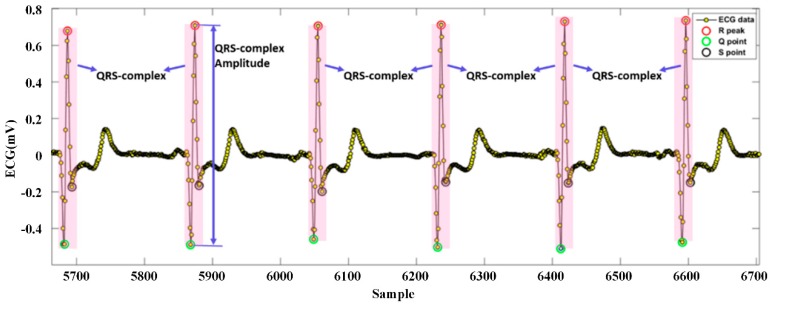
Example of QRS recognition.

An ECG wave consists of many feature points, such as the QRS wave and its onset and offset, and the T wave. Each feature point might be significant for diagnosis. To evaluate the ECG signal and determine the optimal electrode location for wearable single-lead ECG monitoring devices, we must extract the QRS wave from the processed ECG. The R peak is relatively easy to identify, because it usually has the maximum value in the ECG waveform and the largest slope value. Additionally, the R peak is the basis of the detection of other features. We used the slope threshold detection method for QRS recognition [28], of which the block diagram is presented in Figure 10. Searching the local minimum points forwards and backwards from the R wave, we could find the adjacent Q and S points, respectively. Figure 11 showed an example of QRS recognition. The R peak, Q point, and S point were all detected.

#### 2.3.4. Electrode Location Evaluation

As mentioned in Section 2.3.1, three metrics, i.e., SNR, average QRS amplitude, and CV of QRS amplitude, were derived to evaluate ECG signal quality after data acquisition. SNR could be obtained readily after eliminating baseline wandering and noise from the raw ECG data. The QRS amplitude described the strength of the ECG signal, and we calculated the CV of QRS amplitude to compare different electrode pairs regardless of individual differences [16].

To determine the optimal electrode location for wearable single-lead ECG monitoring devices, we first used the SNR and the average QRS amplitude to find the ROI in the coarse-grained stage, that is, the ROI was defined as a square with a comprehensive consideration for the SNR and the QRS amplitude among six-channel ECGs acquired by a WFEES module from the nine coarse-grained squares, as the SNR was the primary metric. Then in the fine-grained stage, we would further combine the CV of QRS amplitude to find the optimal electrode location. Among 54 ECG channels from ROI and eight squares around it, nine candidate channels, i.e., three channels for the highest SNR, three channels for the largest average QRS amplitude, and three channels for the lowest CV, respectively, would be selected. And the optimized electrode locations would be the ECG channels that were selected the most times.

## 3. Results

### 3.1. Improvement of WFEES Module and LANS Method

In this section, we first analyzed the performance enhancement provided by applying the WFEES modules and LANS method. A comparison of the WFEES modules and the BSPM systems is provided in Table 2. As explained in Section 2.2.2, since the maximum value of *A* was 20, and typically, *N* would not be greater than *A* when *A* was larger than 8 for the consideration of testing cost, the WFEES modules required fewer electrodes, while they could measure a reasonable number of ECG lead data sets, like the BSPM system. Additionally, the WFEES module was small, wireless, and wearable, making it more convenient and comfortable for test subjects than the BSPM systems, especially for long-term ECG analysis.

Moreover, a comparison of the direct testing method and the LANS method is listed in Table 3. Considering analysis of ECGs from all 55 squares of the human body, the worst case of the testing lead number for the LANS method was 168, while the testing lead number for direct testing was 330, which was about twice the amount of the LANS method. As for the measurement complexity, the worst case for the LANS method was ⌈28/*N*⌉, which was about half as much for direct testing. The result indicated that the proposed LANS method could reduce the testing lead number, as well as the measurement complexity, effectively.

### 3.2. Optimized Electrode Location

#### 3.2.1. Coarse-Grained Stage for ROI Evaluation

**Figure 12:**
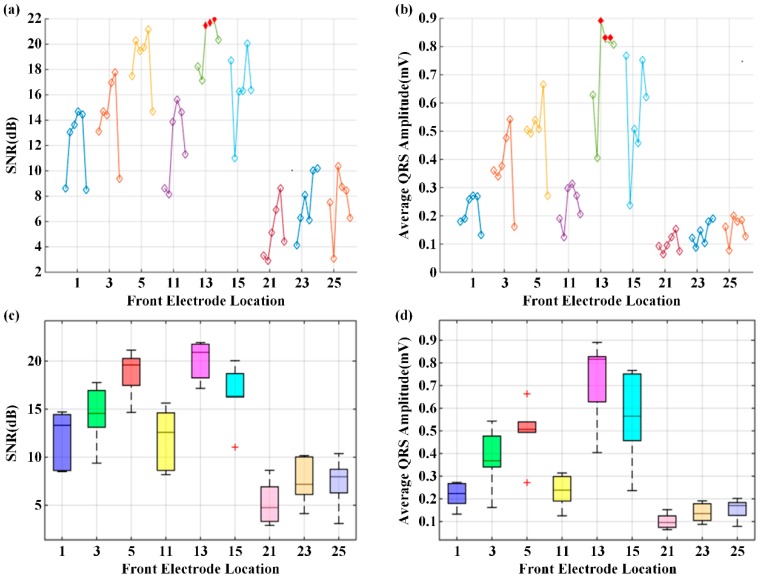
The results of region of interest (ROI) determination: (**a**) Signal-to-noise ratio (SNR) of six channels; (**b**) Average QRS amplitude of six channels; (**c**) SNR distribution of coarse-grained squares; (**d**) QRS amplitude distribution of coarse-grained squares.

Here, we present the results of the case study for determining the optimal electrode location using a WFEES module with the L91S method. Figure 12 illustrates the results of ROI determination considering the SNR and average QRS amplitude. The ECGs were obtained from nine coarse-grained squares, i.e., squares 1, 3, 5, 11, 13, 15, 21, 23, and 25 in Figure 7, on the front side of the 19 subjects’ bodies. In each square location, there were six single-lead ECG channels, indicated as six hollow diamonds on each short line. The six hollow diamonds represented channels 1 to 6 in Figure 1, moving to the right in each short line.

**Figure 13:**
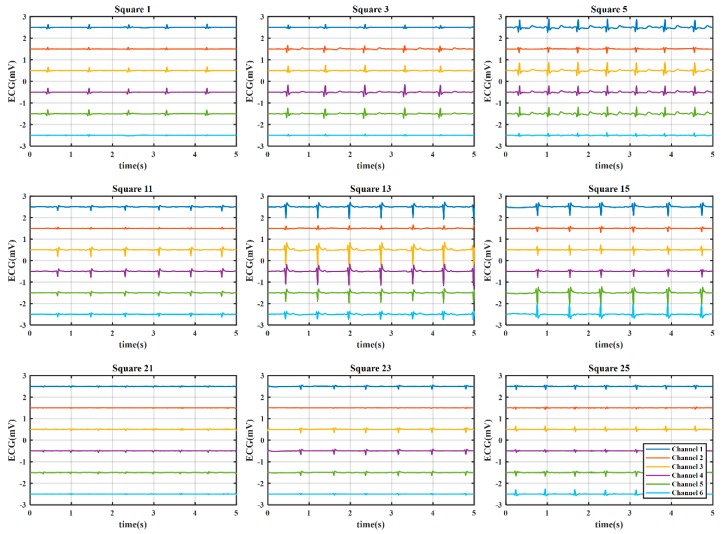
The typical six-channel ECG signals of the coarse-grained squares on the human body. The offset between the adjacent channels was 1 mV, and the vertical ordinate only indicated the relative value.

From Figure 12a,b, we could see that the square 13 provided the highest SNR and average QRS amplitude, particularly in channels 3, 4, and 5, as indicated with filled and red diamonds. To accurately determine the ROI, the distributions of SNR and average QRS amplitude for the nine coarse-grained squares were further presented in Figure 12c,d, respectively. On each box in these figures, the central mark was the median, the edges of the box were the 25th and 75th percentiles, the whiskers extended to the most extreme data points not considered outliers, and outliers were plotted individually. It is obvious that both SNR and average QRS amplitude of square 13 had the best distributions. Figure 13 presents the typical six-channel ECG signals of the coarse-grained squares on the human body. We could also find by observation that the quality of ECG signals from square 13 was relatively better than those from other squares. Therefore, square 13 was determined to be the ROI in our case study.

#### 3.2.2. Fine-Grained Stage to Determine the Optimal Electrode Location and Orientation

**Figure 14:**
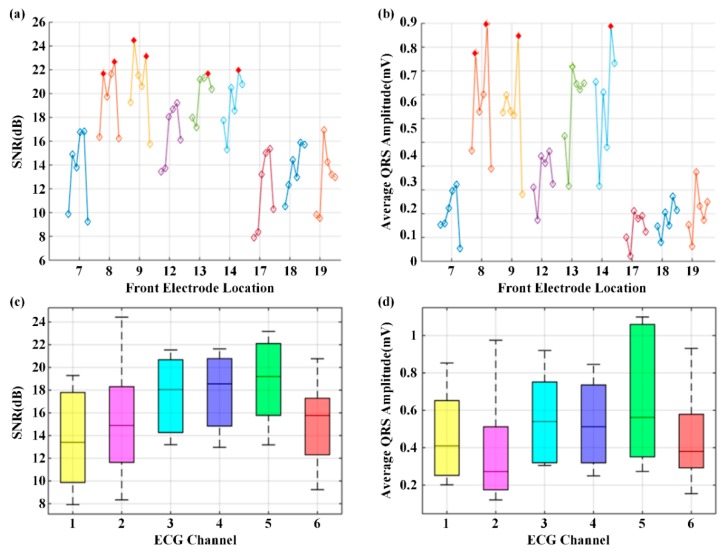
The results of the optimal location and orientation determination: (**a**) Average SNR value; (**b**) Average QRS amplitude; (**c**) SNR distribution; (**d**) QRS amplitude distribution.

**Figure 15:**
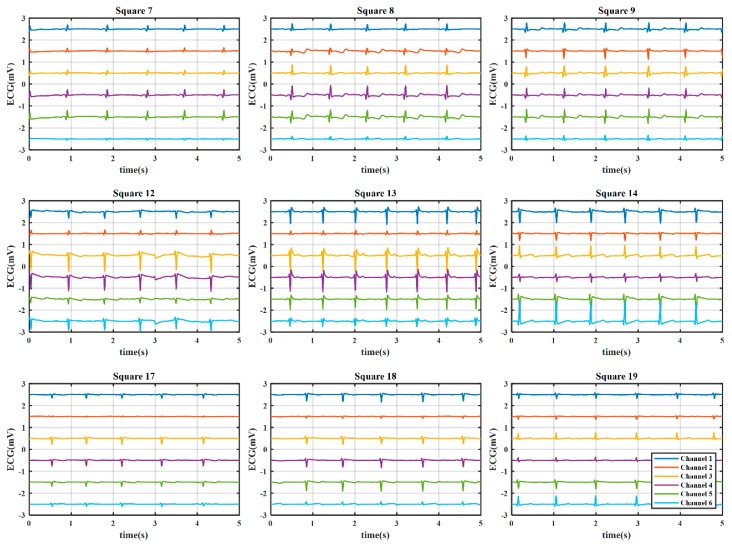
The typical six-channel ECG signals of the fine-grained squares on the human body. The offset between the adjacent channels was 1 mV, and the vertical ordinate only indicated the relative value.

After determining square 13 as the ROI, eight fine-grained squares, i.e., squares 7, 8, 9, 12, 14, 17, 18, and 19, were tested using the WFEES module. Figure 14 shows the results of the SNR and the average QRS amplitude of nine squares in the fine-grained stage for the optimal location and orientation determination. The SNR and the average QRS amplitude were higher than those of squares in the coarse-grained stage. The single-lead electrode pairs with an SNR value larger than 22 dB and an average QRS amplitude larger than 0.9 mV are marked with filled and red diamonds in Figure 14a,b, respectively. From these two figures, channels 2 and 5 were the candidates of the best channel. To further determine which channel is the optimal option, the QRS amplitude and SNR distributions of the six channels were shown in Figure 14c,d, respectively. From the two figures, the SNR and the average QRS amplitude of channel 5 had the best distributions. Therefore, channel 5 was the best channel. The typical six-channel ECG signals of the fine-grained squares on the human body are shown in Figure 15.

**Figure 16:**
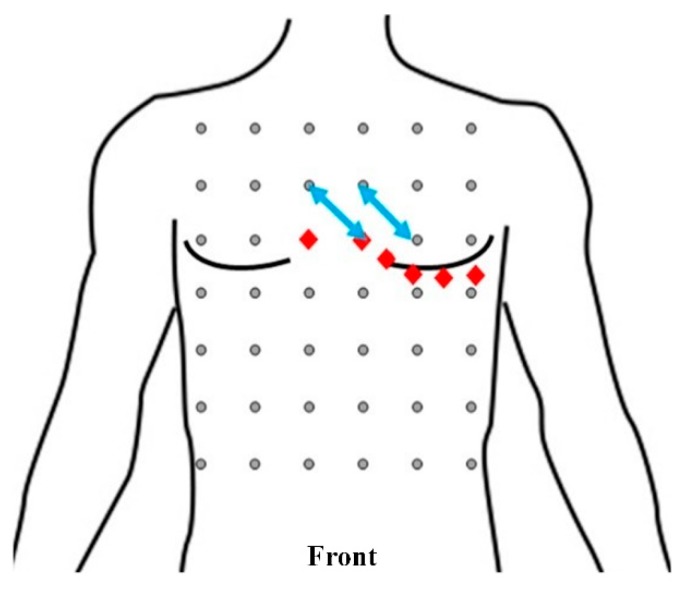
Front view of the positions and orientations of electrode pairs 10/17 and 9/16 (blue arrows). The red diamonds indicate the electrode locations of precordial leads V1–V6 of the standard 12-lead ECG system.

Then, in order to find the optimal location, we listed the average SNR value, the average QRS amplitude, and the CV of the QRS amplitude of channel 5 of each fine-grained square in Table 4. In this case study, the average SNR value that was larger than 21 dB and the average QRS amplitude that was larger than 1 mV were regarded as good. From this table, when taking SNR value into consideration, the SNR values of electrode pairs 9/16, 10/17, and 22/29 were the top three leads. When considering the average QRS amplitude, the best electrode pairs were 9/16, 10/17, and 22/29. As mentioned, since the standard deviation and QRS amplitude were dependent, standard deviation alone could not be used to determine the optimal channel. Thus, the CV was derived. From the table, the lowest three CV values were from electrode pairs 8/15, 10/17, and 27/34. Therefore, by considering SNR, average QRS amplitude, and CV, the optimal channel was electrode pair 10/17, which was above the chest electrodes of standard precordial leads V2 to V4. Furthermore, electrode pair 9/16, which was above the chest electrodes of standard precordial leads V1 and V2, was a channel with good performance considering the three evaluation metrics. These two leads are marked by the blue arrows in Figure 16.

## 4. Discussion

As shown in Section 3.1, the WFEES modules and the LANS method provided an efficient way to investigate ECGs under wearing conditions with a low system cost. As a matter of fact, the WFEES module could be configured to measure other bioelectrical signals through adjusting the electrode distance, the passband of hardware filter, and the sampling rate according to the acquisition requirements of a specific signal. The bottom line is that the combination of the WFEES modules and the LANS method could reduce the comprehensive testing cost of bioelectrical signals effectively and, more importantly, introduce the influences of wearing conditions into the research on these signals, where it was not available in the BSPM systems.

In the case study, to determine the optimized location and orientation for single-lead ECG monitoring devices, we collected ECG data and analyzed them in terms of three metrics: SNR of the signal, average QRS amplitude, and CV of QRS amplitude. According to the experiments, the best locations and orientations for single-lead ECG monitoring devices to collect ECG signals for arrhythmia diagnosis were electrode pairs 10/17 and 9/16, as marked by blue arrows in Figure 16. Electrode pair 10/17 was above the chest electrodes of standard precordial leads V2 to V4. Electrode pair 9/16 was above the chest electrodes of standard precordial leads V1 and V2.

Although we introduced the CV of QRS amplitude as an ECG evaluation metric to eliminate the impact of individual differences on optimized electrode location determination, a total study population of 19 was still relatively small. More subjects of different ages and genders could help balance the distribution of study population and improve the universality of results. Meanwhile, in this case study, we only tested healthy subjects to analyze their ECG signals. Patients with different health profiles should be considered in future experiments, since the ECG waveforms of abnormal heart activities could be different to those of the normal ones. Nevertheless, abnormal ECG beats generally account for a small percentage of resting ECGs, even for primary care population with CVDs [29].

Also, from a medical point of view, an ECG waveform consists of many specific waves, such as the QRS wave, the P wave, and the T wave. Each wave could play a specific role in a given medical analysis. For example, the P wave is often used to detect ventricular hypertrophy, the QRS wave is used to classify cardiac arrhythmias, and the T wave could reflect hyperkalemia. In this case study, we focused on the QRS wave; thus, the P wave, T wave, and other feature points of the ECG waveform were not considered. Hence, the optimized location that we found might be good for detecting arrhythmias, yet not suitable for diagnosing other heart diseases. In future, we will introduce metrics regarding various ECG features to find the optimized electrode locations for other clinical applications based on the ECG signal.

## 5. Conclusions

We designed a wearable ECG investigation tool named the WFEES module and proposed a novel LANS method to simplify the ECG collection and analysis process based on WFEES modules. Compared to the BSPM systems, the WFEES module was wearable and wireless with lower system size and cost, and the proposed LANS method could effectively reduce the measurement complexity. To further evaluate our system and method, we conducted a case study to find the optimized electrode location and orientation for wearable single-lead ECG monitoring devices. Based on a WFEES module, 102 set of single-lead ECG data from 19 healthy subjects were tested to evaluate the optimal electrode locations using the L91S method. The results showed that a single-lead electrode pair should be placed above the chest electrodes of standard precordial leads V1 to V4 to obtain a reliable ECG signal for arrhythmia detection. Additionally, the best orientation was diagonally sloping downwards to the left, as the principal diagonal. This was to be expected because that direction was parallel to the electrical axis of the heart. In the case study, we focused on the SNR and QRS waves, which were useful for detecting arrhythmias. In future, we will consider the P wave, the T wave, and other feature points of the ECG waveform, since each feature peak or wave could play a specific role in a given medical analysis and clinical application.

## Figures and Tables

**Table 1:** Key parameters of the WFEES system.

Parameter	Value
Electrode Distance (cm)	5
Gain (V/V)	1000
Frequency Range (Hz)	0–150
Sampling Rate (Hz)	200
Resolution (bit)	10
Wireless Protocol	BLE

**Table 2:** Comparison of the body surface potential map (BSPM) systems and the WFEES modules.

System	Electrode Number	Testing Lead Number	Lead Form	Data Transfer	System Size	System Cost
BSPM [21]	n/a	192	Wire required	Wired	Large	High
BSPM [22]	120	120	Wire required	Wired	Large	High
BSPM [23]	120	113	Wire required	Wired	Large	High
WFEES	*N* × 4	*N* × 6	Wireless	Wireless	Small	Low

**Table 3:** Comparison of the direct testing and the proposed LANS method.

Method	Testing Lead Number	Measurement Complexity ^1^
Direct Testing	330	⌈55/*N*⌉
LANS	(*A* + 8) × 6	⌈(*A* + 8)/*N*⌉

^1^ “⌈ ⌉” referred to the ceiling function.

**Table 4:** Comparison of channel 5 among the nine squares in Fine-grained step.

Front Electrode Location	Electrode Pair	Average SNR (dB) ^1^	Average QRS Amplitude (mV) ^1^	CV of QRS Amplitude (%)
Square 7	8/15	16.82 ± 4.75	0.42 ± 0.17	0.400
Square 8	9/16	22.64 ± 3.67	1.10 ± 0.53	0.485
Square 9	10/17	23.17 ± 2.25	1.05 ± 0.45	0.428
Square 12	20/27	19.20 ± 5.28	0.56 ± 0.42	0.745
Square 13	21/28	21.66 ± 3.09	0.82 ± 0.38	0.468
Square 14	22/29	21.93 ± 5.47	1.08 ± 0.71	0.653
Square 17	26/33	15.39 ± 4.11	0.29 ± 0.20	0.706
Square 18	27/34	15.91 ± 3.28	0.37 ± 0.16	0.432
Square 19	28/35	13.18 ± 6.01	0.27 ± 0.14	0.508

^1^ The values are presented in the form of “mean ± standard deviation”.
